# Unbiased Identification of Angiogenin as an Endogenous Antimicrobial Protein With Activity Against Virulent *Mycobacterium tuberculosis*

**DOI:** 10.3389/fmicb.2020.618278

**Published:** 2021-01-18

**Authors:** Reiner Noschka, Fabian Gerbl, Florian Löffler, Jan Kubis, Armando A. Rodríguez, Daniel Mayer, Mark Grieshober, Armin Holch, Martina Raasholm, Wolf-Georg Forssmann, Barbara Spellerberg, Sebastian Wiese, Gilbert Weidinger, Ludger Ständker, Steffen Stenger

**Affiliations:** ^1^Institute of Medical Microbiology and Hygiene, University Hospital Ulm, Ulm, Germany; ^2^Core Unit Mass Spectrometry and Proteomics, Ulm University, Ulm, Germany; ^3^Core Facility of Functional Peptidomics, Ulm University, Ulm, Germany; ^4^Institute of Biochemistry and Molecular Biology, Ulm University, Ulm, Germany; ^5^Pharis Biotec GmbH, Hannover, Germany

**Keywords:** antimicrobial peptide, *Mycobacterium tuberculosis*, endogenous protein, antibacterial, human

## Abstract

Tuberculosis is a highly prevalent infectious disease with more than 1.5 million fatalities each year. Antibiotic treatment is available, but intolerable side effects and an increasing rate of drug-resistant strains of *Mycobacterium tuberculosis* (*Mtb*) may hamper successful outcomes. Antimicrobial peptides (AMPs) offer an alternative strategy for treatment of infectious diseases in which conventional antibiotic treatment fails. Human serum is a rich resource for endogenous AMPs. Therefore, we screened a library generated from hemofiltrate for activity against *Mtb*. Taking this unbiased approach, we identified Angiogenin as the single compound in an active fraction. The antimicrobial activity of endogenous Angiogenin against extracellular *Mtb* could be reproduced by synthetic Angiogenin. Using computational analysis, we identified the hypothetical active site and optimized the lytic activity by amino acid exchanges. The resulting peptide-Angie1-limited the growth of extra‐ and intracellular *Mtb* and the fast-growing pathogens *Escherichia coli*, *Pseudomonas aeruginosa*, and *Klebsiella pneumoniae*. Toward our long-term goal of evaluating Angie1 for therapeutic efficacy *in vivo*, we demonstrate that the peptide can be efficiently delivered into human macrophages *via* liposomes and is not toxic for zebrafish embryos. Taken together, we define Angiogenin as a novel endogenous AMP and derive the small, bioactive fragment Angie1, which is ready to be tested for therapeutic activity in animal models of tuberculosis and infections with fast-growing bacterial pathogens.

## Introduction

Tuberculosis is among the top 10 causes of death and the leading cause from a single infectious agent (World Health Organization, 2019). Even though tuberculosis is a treatable and curable disease, conventional antibiotic therapy may fail. Major obstacles in the treatment are non-adherence of patients to the 6-months therapy, intolerable side effects, and the emergence of drug resistant strains of *Mycobacterium tuberculosis* (*Mtb*). WHO estimates that approximately 350 new cases of multidrug-resistant tuberculosis (MDR-TB) occur each year and declared MDR-TB as a public health crisis, which requires global attention by health care authorities. Second-line drugs are available for the treatment of MDR-TB but they are expensive, toxic, and require at least 12 months of therapy with cure rates below 60% (World Health Organization, 2019). Therefore, new therapeutic strategies are desperately needed. Ideally, new compounds would attack the bacteria by mechanisms distinct from conventional antituberculotic drugs, which mostly act by inhibiting bacterial RNA or protein synthesis. An attractive approach is the development of antimicrobial peptides (AMP), which are small peptidic compounds (<10 kDa) that are highly diverse in length, sequence, structure, and activity (Wang, 2014). AMPs are widespread in nature and are also part of the human innate immune system. Endogenous AMPs with activity against virulent *Mtb*, include Granulysin, Cathelicidin (LL-37), ß-Defensins, Cecropin, Lipocalin 2, or Teixobactin (Gutsmann, 2016). Studies on the mechanisms of action of these AMPs are limited since *Mtb* has a notoriously low metabolism, long generation time and is a biosafety level 3 pathogen. Imaging of AMP-treated *Mtb* indicates that Granulysin and LL-37 disrupt the mycobacterial cell wall (Stenger et al., 1998; Deshpande et al., 2020). It is unclear whether this is the lethal hit or only the initial step to allow the entry into the bacterial cytosol to reach its final intracellular target. The advantages of endogenous AMPs for the treatment of infectious diseases include the easy synthesis and the limited propensity to induce toxicity, allergic reactions, or drug resistance (Gutsmann, 2016). Recently, we developed a standardized protocol to generate libraries of highly concentrated endogenous peptides from human bodily fluids (Schulz-Knappe et al., 1997; Bosso et al., 2018). Starting from thousands of liters of hemofiltrate, a waste product of hemodialysis of patients with renal failure, a peptide library was generated. Peptides were separated into 300–500 pools based on charge (cation-exchange separation) and hydrophobicity (reversed-phase liquid chromatography). This hemofiltrate peptide library is a salt free source of highly concentrated peptides and small proteins (<30 kDa), which can be exploited for the unbiased search for antimicrobial peptides (Bosso et al., 2018). Here, we screened the pools of the peptide library for activity against virulent *Mtb*. We identified the 14.1 kDa protein Angiogenin, which activates several signaling transduction pathways in eukaryotic cells thereby affecting physiological and pathological processes (Sheng and Xu, 2016). We chemically modified Angiogenin for size, solubility, and activity, yielding “Angie1.” Angie1 is not only active against extracellular *Mtb*, but importantly also enters macrophages, the major host cell of mycobacteria, where it limits mycobacterial proliferation.

## Materials and Methods

### Generation of a Hemofiltrate Peptide Library and Further Purification of the Active Molecule

A peptide bank (HF040823) prepared from 10 L hemofiltrate was generated by combining stepwise pH (from 3.6 to 9) elution in cation-exchange chromatography with reversed-phase chromatographic fractionation of every pH pool as described (Schulz-Knappe et al., 1997). Further bioassay-guided purification was performed by reversed-phase liquid chromatography on Source 15 (GE Healthcare, Buckinghamshire, United Kingdom) and Aqua C18 (Phenomenex, Aschaffenburg, Germany) columns. Aliquots of these peptide bank fractions were lyophilized and used for testing the antimicrobial activity against extracellular *M. tuberculosis* as mentioned below.

### Mass Spectrometry Identification of the Active Molecule

Intact mass measurement was performed on a REFLEX III MALDI-TOF mass spectrometer (Bruker-Daltonics, Billerica, MA, United States) in linear mode (Rodríguez et al., 2018). Sequencing of proteolytic fragments was performed as previously described (Groß et al., 2020). Briefly, reduction with DTT (5 mM), carbamidomethylation with iodoacetamide (50 mM) and trypsin-digestion were performed prior to analysis on an Orbitrap Elite™ Hybrid Ion Trap-Orbitrap Mass Spectrometer coupled to an U3000 RSLCnano uHPLC (Thermo Fisher Scientific, Waltham, MA, United States). Databases were searched using PEAKs X+ studio (Zhang et al., 2012). For peptide identification, MS spectra were correlated with the UniProt human reference proteome set. Theoretical average molecular masses were calculated with ProtParam.

### Identification of Antimicrobial Regions in Angiogenin

Antimicrobial regions of Angiogenin were predicted by an automated web server (AMPA; Torrent et al., 2012) followed by determination of antimicrobial sequences using the database CAMPR3 (Waghu et al., 2016).

### Synthesis of Angiogenin and Angie1

Angiogenin, Angie1, and Atto647N-labeled Angie1-Atto647N were obtained from PSL Heidelberg (PSL, Heidelberg, Germany) using F-moc chemistry. For selected experiments Angie1 was synthesized on site (CFP, Ulm, Germany) on a Liberty blue peptide synthesizer (CEM, Kamp-Lintfort, Germany). All peptides were purified to >95% homogeneity by reversed-phase HPLC and diluted in aqua ad iniectabilia (Ampuwa, Fresenius Kabi) prior to use. Composition of peptides was confirmed by amino acid analysis and mass spectrometry as described (Ständker et al., 1998). Specifically, the absence of additional peptides in the Angie1 preparation was confirmed by reverse phase HPLC (Supplementary Figure S1).

### Bacteria

*Mycobacterium tuberculosis* H37Rv (ATCC 27294) was grown in suspension with constant gentle rotation in roller bottles (Corning, Corning, NY, United States) containing Middlebrook 7H9 broth (BD Biosciences, Franklin Lakes, NK, United States) supplemented with 1% glycerol (Roth, Karlsruhe, Germany), 0.05% Tween 80 (Sigma-Aldrich, Steinheim, Germany), and 10% Middlebrook oleic acid, albumin, dextrose, and catalase enrichment (BD Biosciences, OADC). Aliquots from logarithmically growing cultures were frozen at −80°C in 7H9 broth with 20% glycerol, and representative vials were thawed and enumerated for viable colony forming units (CFU) on Middlebrook 7H11 plates (BD Biosciences). Staining of bacterial suspensions with fluorochromic substrates differentiating between live and dead bacteria (BacLight, Invitrogen, Carlsbad, CA, United States) revealed a viability of the bacteria >90%. Thawed aliquots were sonicated in a water bath for 10 min at 40 kHz and 110 W before use. *Pseudomonas aeruginosa* (ATCC 27853), Extended Spectrum Beta-Lactamase (ESBL) *Klebsiella pneumoniae* (ATCC 7000603), and *Escherichia coli* DH5*α* (Law and Kelly, 1995) were cultured in liquid THY broth (Oxoid, ThermoFisher Scientific, Schwerte, Germany) supplemented with 0.5% yeast extract (BD Biosciences) at 37°C overnight in a 5% CO_2_ atmosphere.

### Generation of Human Monocyte Derived Macrophages

Human peripheral blood mononuclear cells (PBMC) were isolated by density gradient centrifugation (Ficoll-Paque™ Plus; GE Healthcare) of buffy coat preparations from anonymous donors (Institute of Transfusion Medicine, Ulm University). Monocytes were isolated from PBMCs by adherence on tissue culture treated plastic flasks (Falcon, Corning, NY, United States). Monocyte-derived macrophages were generated by incubation with granulocyte-macrophage colony-stimulating factor (10 ng/ml; Miltenyi Biotec, Bergisch Gladbach, Germany) in Macrophage-Serum Free Medium (M-SFM; Gibco, ThermoFisher Scientific, Schwerte, Germany) for 6 days. Phenotypic characterization by flow cytometry demonstrated that macrophages expressed CD68 (anti–CD68-FITC, clone Y1/82A; BD Biosciences, Franklin Lakes, NJ, United States) and MHCII (anti–HLA-DR-PerCP, clone L243; BD Biosciences) as described (Bruns et al., 2012).

### ^3^H-Uracil Proliferation Assay

As a correlate of mycobacterial metabolism, we measured the rate of RNA synthesis by determining the uptake of radioactively-labeled 5.6-^3^H-Uracil (ART-0282, Biotrend, Cologne, Germany). About 2 × 10^6^ sonicated *Mtb* were incubated with peptides in a 96-well plate in RPMI 1640 diluted 1:4 in distilled water and supplemented with 2 mM L-glutamine (PAN biotech, Aidenbach, Germany), 10 mM sodium bicarbonate (NaHCO_3_, Roth), as previously described (Liu et al., 2006). The final volume was 100 μl and all samples were set up in triplicates. After 72 h, ^3^H-Uracil (0.3 μCi/well) was added and cultures were incubated for additional 18 h. *Mtb* were inactivated by 4% PFA for 20 min, followed by a transfer onto glass fiber filters (Printed Filtermat A, PerkinElmer, Waltham, MA, United States) using a 96-well-cell harvester (Inotech, Nabburg, Germany). Fiber filters were sealed at 75°C with a wax plate containing the scintillation liquid (MeltiLex, Perkin Elmer). Incorporation of ^3^H-Uracil by the bacilli was measured using a ß-counter (Sense Beta, Hidex, Turku, Finland). Antimicrobial activity (%) was calculated as follows: (cpm of the treated sample)/(cpm of the un-treated sample) × 100.

### Activity of Angie1 Against Fast-Growing Bacteria

The activity of Angie1 against fast-growing bacteria was determined by radial diffusion assay. Overnight cultures of bacteria were suspended in 10 mM sodium phosphate buffer (Merck) and optical density was determined spectrophotometrically (UV-1800, Shimadzu Corporation) at 600 nm (OD_600_). An OD_600_ value of 1.0 equals 8 × 10^8^ cells/ml. After dilution, 2 × 10^7^ bacteria were seeded into a petri dish in 1% agarose (Sigma-Aldrich) dissolved in 10 mM sodium phosphate buffer. Plates were cooled and cavities with a diameter of 2–3 mm were placed into the agar. Angie1 was diluted in 10 μl ddH_2_O to its final concentration and added into the cavities. Following incubation at 37°C in ambient air for 3 h, plates were overlaid with 10 ml of a 1% agarose solution containing 3% Tryptone Soy broth (Oxoid) dissolved in 10 mM phosphate buffer. The diameters of the inhibition zones were determined following 16–18 h incubation at 37°C in a 5% CO_2_ atmosphere.

### Quantification of Intracellular Mycobacterial Growth

Macrophages were infected in six-well plates with single-cell suspensions of *Mtb* [multiplicity of infection (MOI) = 5] in macrophage-serum free medium. After 2 h, macrophages were washed thoroughly to remove extracellular *Mtb* and harvested using EDTA (1 mM). The rate of infection and cellular viability were determined using Auramine-Rhodamine (Merck) and Annexin V staining (BD, Franklin Lakes, NJ, United States) as previously described (Bruns et al., 2012). The rate of infected macrophages was donor-dependent and ranged between 25 and 40%. About 2 × 10^5^ infected macrophages were seeded in 24-well plates and incubated with Angiogenin (10 μM; PSL, Heidelberg, Germany) or Angie1 (27, 54, and 108 μM; CFP, Ulm, Germany) for 4 days. To enumerate the number of viable bacilli, infected macrophages were lysed with 0.3% saponin (Sigma-Aldrich). Cell lysates were vigorously resuspended, transferred in screw cap tubes and sonicated for 10 min. Afterward, serial dilutions (1:10; 1:100; and 1:1000) of the sonicates were plated on 7H11 agar plates (BD) and incubated for 21 days before counting the CFU.

### Toxicity of Peptides Against Macrophages

About 1 × 10^5^ macrophages were incubated with peptides for 18 h in a 96-well plate, followed by addition of 10% PrestoBlue™ (Thermo Fisher) for 20 min. The non-fluorescent resazurin-based PrestoBlue is reduced to fluorescent resorufin by mitochondrial enzymes of viable cells (Xu et al., 2015). The fluorescence intensity (FI) was measured at *λ*_ex_ 560 nm and λ_em_ 600 nm using Infinite 200 PRO (Tecan, Männedorf, Suisse) plate reader. Cell viability (%) was calculated using the following formula where the ratio of the FIs of the sample and the untreated control is calculated after subtracting the background FI introduced by the same volume of cell culture medium: [FI (sample)-FI (background)]/[FI (untreated control)-FI (background)] × 100.

### Toxicity of Angie1 Against Zebrafish Embryos

Wild-type zebrafish embryos were dechorionated at 24 h post fertilization (hpf) using digestion with 1 mg/ml pronase (Sigma) in E3 medium (83 μM NaCl, 2.8 μM KCl, 5.5 μM CaCl_2_, and 5.5 μM MgSO_4_). Embryos were exposed for 24 h, in groups of three, to 100 μl of E3 medium containing Angie1 at 1, 10, and 100 μM. Each concentration was tested in two independent assays, each of which was performed on 10 × 3 embryos. The peptide solvent (PBS), diluted in E3, was used as negative control at the same amount as introduced by the highest peptide concentration. As positive control for acute toxicity/cytotoxicity the pleurocidin antimicrobial peptide NRC-03 (GRRKRKWLRRIGK-GVKIIGGAALDHL-NH2) was used at a concentration of 6 μM as described (Morash et al., 2011). Abamectin at a concentration of 3.125 μM was used as positive control for neurotoxicity (Raftery et al., 2014). At 48 hpf (after 24 h of incubation) embryos were scored in a stereomicroscope for signs of acute toxicity/cytotoxicity (lysis and/or necrosis), developmental toxicity (delay and/or malformations), or cardiotoxicity (heart edema and/or reduced or absent circulation). Each embryo was also touched with a needle and reduced or absent touch response (escape movements) was evaluated as signs of neurotoxicity if and only if no signs of acute toxicity were present in the same embryo. Embryos were categorized within each of these toxicity categories into several classes of severity according to the criteria listed in Supplementary Figure S3A. Chi-Square test was used to calculate whether the distribution of embryos into toxicity classes differed significantly between the PBS negative control and the test substances.

### Half-Life of Angie1 in Human Serum

Human serum (14-490E, Lonza, Basel, Switzerland) was spiked with 10 μM Angie1 and incubated at 37°C for 2 h in triplicates. After 5, 10, 20, 30, 45, 60, 90, and 120 min; aliquots (5 μl) were diluted with 1.5 ml ice cold TFA (0.1%). Around 15 μl of each sample was analyzed using an Orbitrap Elite system (Thermo Fisher Scientific) as described above. XCalibur Qual Browser (Thermo Fisher Scientific) was used for visualization of the total ion chromatograms and spectra, as well as for signal area calculation. The theoretical spectrum of Angie1 and its degradation products were generated and the highest intensity signals were used as reference for detecting Angie1 and its degradation products. Plot graphs (signal area vs. time) and half-lives were calculated with GraphPad Prism 8.0 (GraphPad, La Jolla, CA, United States).

### Generation of Angie1 and Angiogenin-Containing Liposomes

Liposomes were generated by lipid film hydration as described (Kallert et al., 2015). Briefly, dimethyldioctadecylammonium (DDA; 0.3 mg/ml; Avanti Polar Lipids), D-(+)-trehalose 6,6′-dibehenate (TDB; 0.25 mg/ml; Avanti Polar Lipids, Sigma Aldrich) and L-α-phosphatidylcholine (PC; 0.9 mg/ml; Avanti Polar Lipids) were mixed in chloroform (VWR): methanol (Sigma-Aldrich; 9:1, v/v). The organic solvents were evaporated under nitrogen flow. Liposomes were formed by hydrating the lipidfilm in 500 μl 10 mM Tris-buffer (pH 7.4; Sigma-Aldrich) for 25 min at 57°C within between vortexing, as previously described (Kennerknecht et al., 2020). Angie1 or Angiogenin were added in a 1:1 mixture to the liposomes at a final concentration of 2.7 mM. The size of liposomes was determined by Nanoparticle Tracking Analysis (NTA) using a ZetaView TWIN (Particle Metrix, Inning, Germany). Samples were diluted in TRIS-buffer and videos of the light-refracting particles were recorded with the following settings: 25°C fixed temperature, 11 positions, 1 cycle, sensitivity 85, shutter 100, 15 fps, 2 s videos/position, and six measurements. The number and size distribution were evaluated by ZetaView Analyze (Version 08.05.05 SP2), as previously described (Conzelmann et al., 2020).

### Uptake of Angie1 by Macrophages: Flow Cytometry and Confocal Laser Microscopy

For investigation of Angie1 internalization, 1 × 10^6^ macrophages were incubated with Angie1-Atto647N or Angie1-Atto647N Lip for 18 h. Samples were then analyzed using a FACS Calibur (BD Biosciences) with FlowJo v10.4.1 (BD). For confocal laser microscopy, 0.1 × 10^6^ macrophages were seeded in 200 μl M-SFM in an eight-chamber slide (Thermo Fisher) and incubated with Angie1-Atto 647 N (54 μM) or Angie1-Atto 647 N Lip (54 μM) or Angiogenin-Atto647N Lip. After overnight incubation, cells were fixed (4% PFA, Sigma-Aldrich), followed by blocking in 2% BSA. Subsequently macrophages were labeled with major histocompatibility antigen class II antibodies (HLA-DR; 1:200, clone L243, Leinco, St. Louis, MS, United States) detected by Cy2-conjugated goat anti-mouse antibodies (1:200, Dianova, Hamburg, Germany). Cell nuclei were stained with DAPI (1:200, Sigma-Aldrich) diluted in 1% BSA and 0.1% Triton X-100 in PBS (all Sigma-Aldrich). Images were acquired using the inverted laser scanning confocal microscope LSM 710 (Zeiss, Oberkochen, Germany). Image processing was performed using ImageJ software (v 1.52c). All images displayed in this study are processed for brightness/contrast.

### Statistical Analysis

All statistical analyses were performed using GraphPad Prism v8.2.1 (GraphPad Software). A difference in results giving a value of *p* < 0.05 was defined as significant.

### Ethical Statement

Zebrafish embryos were used at stages up to 2 days post fertilization (dpf), which is before they start to feed at 6 dpf. Embryos that do not yet require feeding are not covered by EU and German animal experiment and welfare legislation (§14 TierSchVersV). Embryos were euthanized at the end of the test by rapid freezing, which is considered the most humane method for euthanasia for fish embryos. Adult fish housing and care were approved by the state of Baden-Württemberg and are monitored by Ulm University animal welfare executives and veterinaries of the city of Ulm.

## Results

**Figure 1:**
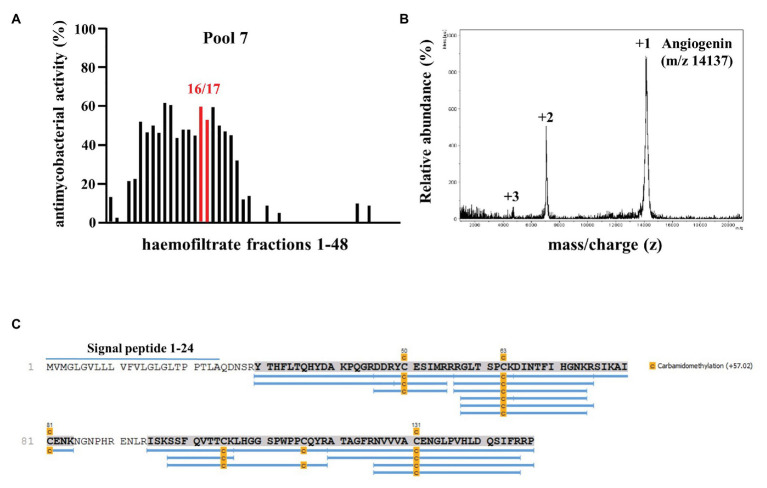
Screening a hemofiltrate library for activity against extracellular *Mycobacterium tuberculosis* (*Mtb*). **(A)** Around 48 fractions (each 10 μl) of pool seven of the hemofiltrate library were incubated for 96 h with extracellular *Mtb*. ^3^H-Uracil was added for the final 24 h of incubation. The uptake of ^3^H-Uracil was measured by scintillation counting in a ß-counter. Antimicrobial activity was calculated by comparison to the untreated control. The bars present the average antimycobacterial activity (%) calculated from triplicates of a single experiment. **(B)** Subfractions 7–16/17 were analyzed *via* mass spectrometry. Spectrum analysis yielded three distinct peaks, identifying Angiogenin with a molecular mass of 14,137 Dalton. **(C)** Angiogenin sequence coverage (88%) by MS/MS analysis of the sample after carbamidomethylation and digestion with trypsin. Full sequence (Angiogenin precursor): signal peptide: 1–24 and Angiogenin 25–147.

**Figure 2:**
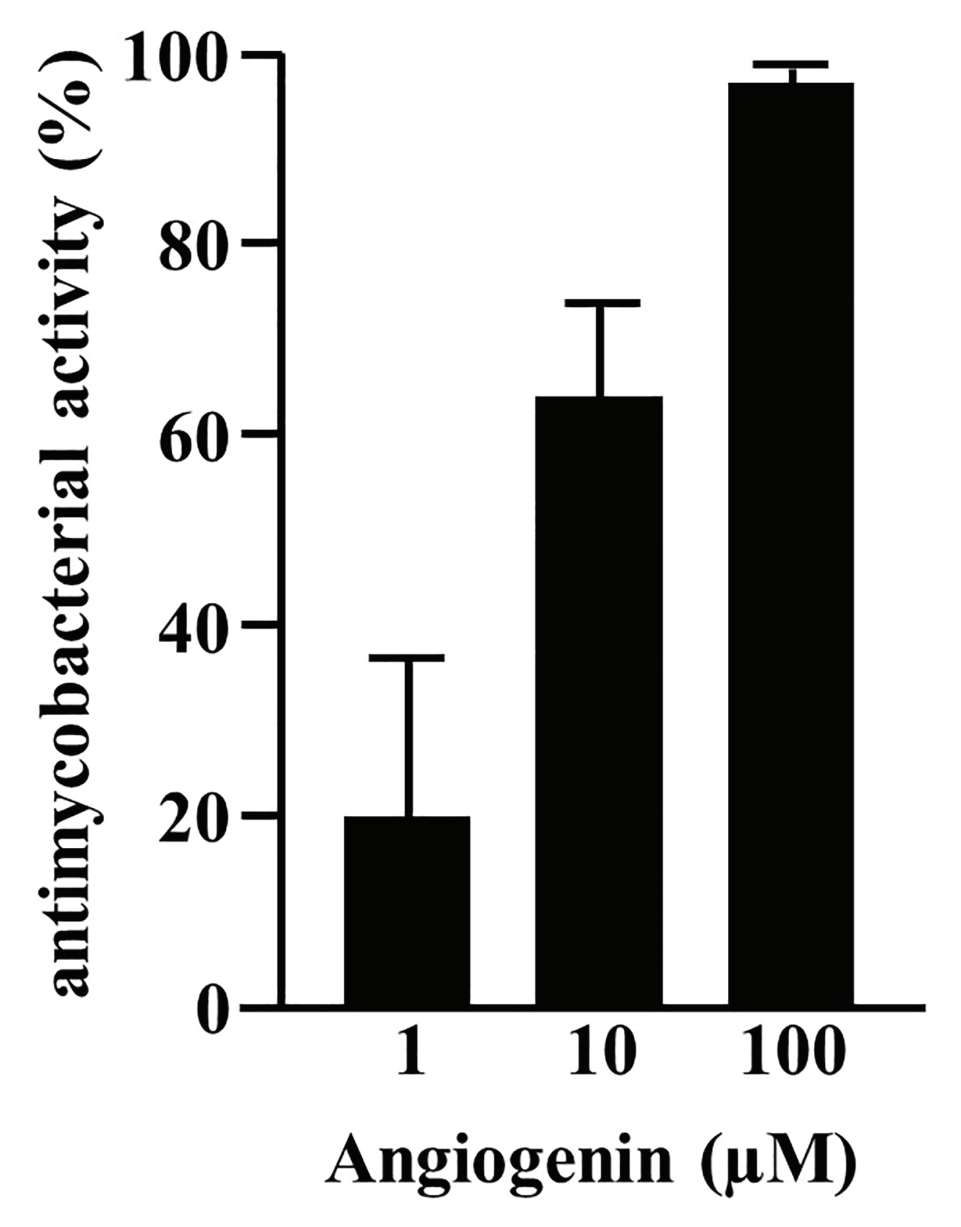
Activity of synthetic Angiogenin against extracellular *Mtb*. About 2 × 10^6^ extracellular *Mtb* were incubated for 96 h with synthetic Angiogenin (PSL Heidelberg). ^3^H-Uracil was added for the final 24 h, of incubation and ^3^H-Uracil uptake was measured by scintillation counting. Antimycobacterial activity was calculated as follows: [(counts/sample: count/untreated control) ×100]. Bars represent the mean antibacterial activity (%) ± SD calculated from triplicates of three independent experiments.

In searching for endogenous AMPs with activity against virulent *Mtb* we screened a peptide library obtained from hemofiltrate as described (Schulz-Knappe et al., 1997). Extracellular bacilli were incubated with three dilutions (1:10, 1:100, and 1:1,000) of these fractions. As a correlate for mycobacterial viability, we measured the incorporation of ^3^H-Uracil after 72 h of incubation. We tested eight pools (separated by charge), each containing 48 fractions (separated for hydrophobicity) from the original hemofiltrate library. Fractions yielding a dose-dependent antimicrobial activity >50%, that were reproducible in at least two experiments, were scored positive (Figure 1A, pool seven, fractions 16/17). After two additional rounds of sub-fractionation, we analyzed the most active fraction by mass spectrometry to identify the bioactive peptide(s). Mass spectrometry yielded three distinct peaks all unequivocally assigned to differently charged Angiogenin (mass 14,137 Da; Figure 1B) after MS/MS sequencing (Figure 1C). To validate the result of the screening, we tested the activity of recombinant Angiogenin against extracellular *Mtb* and found a dose dependent activity peaking at 100 μM (94 ± 2%; Figure 2).

**Figure 3:**
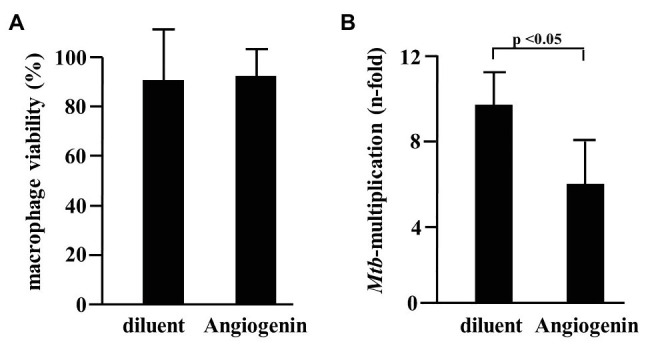
Effect of Angiogenin on macrophage viability and *Mtb* multiplication. **(A)** Macrophages were incubated for 24 h with Angiogenin (10 μM, PSL Heidelberg) or diluent. Macrophage viability was quantified using a PrestoBlue™ assay and is given in % of the un-treated sample. Bars represent the mean viability of macrophages (%) ± SD calculated from triplicates of three independent experiments. **(B)** Macrophages were infected with *Mtb*, followed by incubation with Angiogenin (10 μM) for 4 days. Lysates were plated on 7H11 agar plates and the number of CFU counted after 21 days of incubation. *Mtb*-multiplication was calculated by comparison of the CFU determined at d4 and d0. The graph gives the mean values ± SD of n-fold mycobacterial growth as compared to d0 for six independent donors. Statistical analysis was performed using a paired *t*-test.

**Figure 4:**
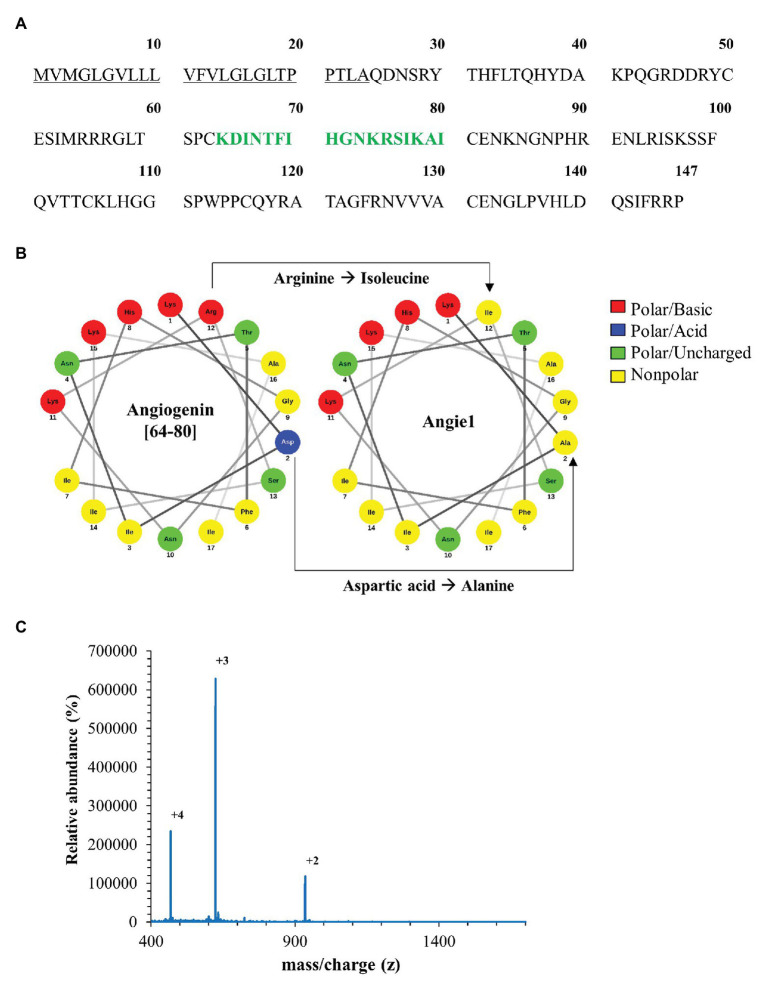
*In silico* design of Angie1 from Angiogenin. **(A)** Sequence of Angiogenin precursor (1–24: signal peptide; 25–147: Angiogenin). The cytolytic active site was determined by AMPA (64–80aa) and is highlighted in green. **(B)** Helical wheel projection of the cytolytic region (left panel) demonstrating the polarity of the amino acids. The right projection shows the modifications of the cytolytic region yielding Angie1. **(C)** Mass spectrometry analysis of Angie1. Angie1 was analyzed by LC-ESI-MSMS (Orbitrap Elite system). The spectrum shows the [M+2H]^2+^, [M+3H]^3+^, and [M+4H]^4+^ multicharged signals with the monoisotopic m/z value.

Since Angiogenin is an endogenous peptide it should not be toxic for human cells. However, to exclude a diluent-mediated toxic effect, we incubated Angiogenin with primary human macrophages and determined cell viability after 18 h. Angiogenin at concentrations active against extracellular *Mtb* (10 μM) did not affect the viability of macrophages as determined by the ability to metabolize resazurin (Figure 3A). After the initial exponential multiplication in the pulmonary parenchyma, *Mtb* infects macrophages where the pathogen can multiply, persist, and cause chronic disease or latent infection. Effective drugs should therefore not only limit the growth of extracellular *Mtb*, but also act on the intracellular pathogen. To test the ability of Angiogenin to limit the growth of intracellular *Mtb*, we infected primary human macrophages with virulent *Mtb* in the presence of the peptide or the diluent (water) only. Angiogenin (10 μM) significantly limited the multiplication of intracellular *Mtb* from 9.5 to 5.2-fold (*p* < 0.05; Figure 3B). These results demonstrate that screening of a hemofiltrate library is a successful strategy to identify novel AMPs. A major challenge for the clinical application of AMPs is the stability of the compound. One strategy to increase the stability is to identify active sequences within the peptide and generate smaller compounds, while maintaining bioactivity. *In silico* databank analysis predicted that amino acids 64–80 of Angiogenin are responsible for the antimicrobial effect (Figure 4A). Since positively charged peptides presumably interact more effectively with the negatively charged mycobacterial cell wall (Gutsmann, 2016), aspartic acid was changed to alanine and arginine was changed to isoleucine to prevent interference of the positively charged arginine and lysine (Figure 4B). The novel peptide “Angie1” contains 16 amino acid residues, has a molecular weight of 1.86 kDa (Figure 4C) and a net charge of +3 and is soluble in water. The presence of degradation products is highly unlikely, since MS revealed distinct peaks depending on the charge. In addition, the experimental monoisotopic mass is 1867.104 Da and almost identical to the theoretical mass (1867.094 Da).

**Figure 5:**
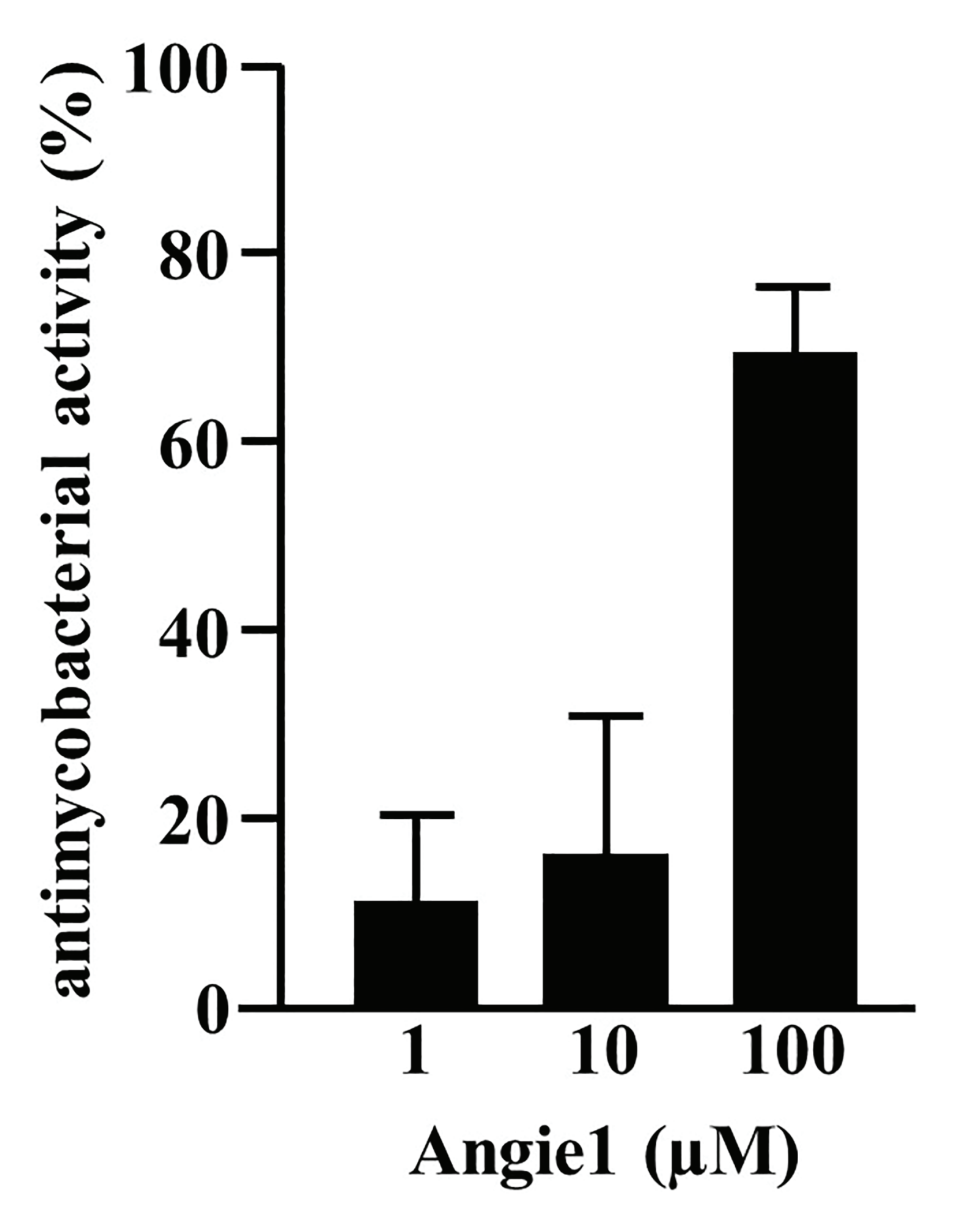
Antimycobacterial activity of Angie1 against extracellular *Mtb*. About 2 × 10^6^ extracellular *Mtb* were incubated for 96 h with Angie1 (CFP). Uptake of ^3^H-Uracil was measured by scintillation counting. Antimicrobial activity was calculated by comparison of treated samples to untreated controls. Bars represent the mean antibacterial activity (%) ± SD calculated from triplicates of three independent experiments.

**Figure 6:**
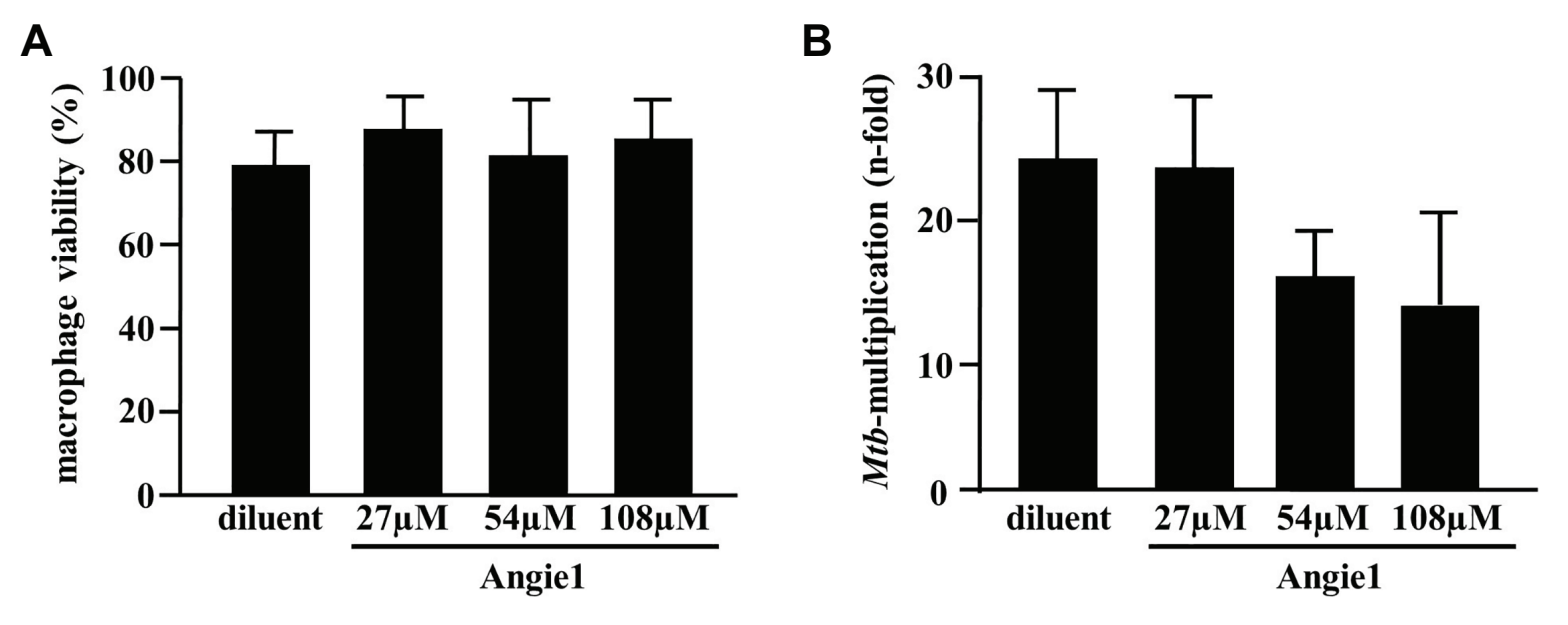
Effect of Angie1 on macrophage viability and multiplication of intracellular *Mtb*. **(A)** Macrophages were incubated with Angie1 (PSL Heidelberg) at indicated concentrations for 24 h and viability was determined by the PrestoBlue™ assay. Bars represent the mean viability of macrophages (%) ± SD calculated from triplicates of three independent experiments. **(B)** Macrophages were infected with *Mtb* followed by incubation with Angie1 for 4 days. *Mtb*-multiplication was determined by plating cell lysates on 7H11 agar plates to determine and counting the number of CFU after 21 days of incubation. The graph gives the mean values ± SD of n-fold mycobacterial growth as compared to d0 for four independent donors.

As predicted, the modified fragment Angie1 maintained the activity against extracellular *Mtb* to a similar extent as the parental Angiogenin (78% at 100 μM; Figure 5). Angie1 was not toxic for primary human macrophages at 27, 54, or 108 μM (Figure 6A) and showed moderate but reproducible activity against intracellular *Mtb*. The antimicrobial activity did not increase at higher concentrations (108 μM; Figure 6B).

**Figure 7:**
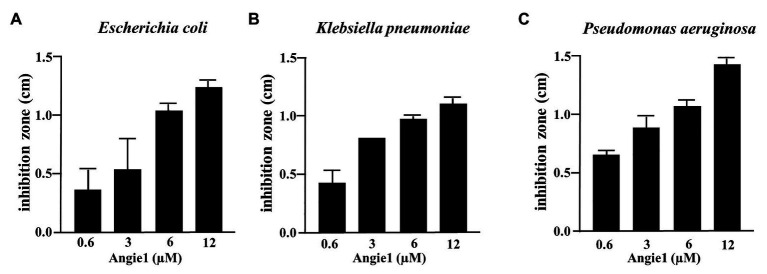
Antimicrobial effect of Angie1 against fast-growing bacteria. About 2 × 10^7^ bacteria were seeded into a petri dish containing agarose. Angie1 (Core Facility Peptidomics) was given into cavities in the agarose. After 3 h, plates were overlaid with agarose and after 18 h of incubation, inhibition zones were measured for **(A)** *Escherichia coli*, **(B)** *Klebsiella pneumoniae* and **(C)** *Pseudomonas aeruginosa*. Bars represent the mean values of inhibition zone size (cm) ± SD calculated from three independent experiments.

One potential advantage of AMPs is the broad spectrum of bacteria on which they act, because they do not target specific metabolic or biochemical pathways like many conventional antibiotics. To determine whether Angie1 also acts on other bacterial species than *Mtb*, we evaluated the activity against three fast-growing bacteria (*E. coli*, *K. pneumoniae*, and *P. aeruginosa*), all of which are relevant pathogens especially in hospital-acquired infections. Angie1 inhibited the growth of all three species in a dose dependent manner at concentrations in the low micromolar range (Figure 7). Taken together our results demonstrate that Angie1 is a novel antimicrobial peptide with activity against extracellular and intracellular *Mtb* and a selection of Gram-negative rods.

**Figure 8:**
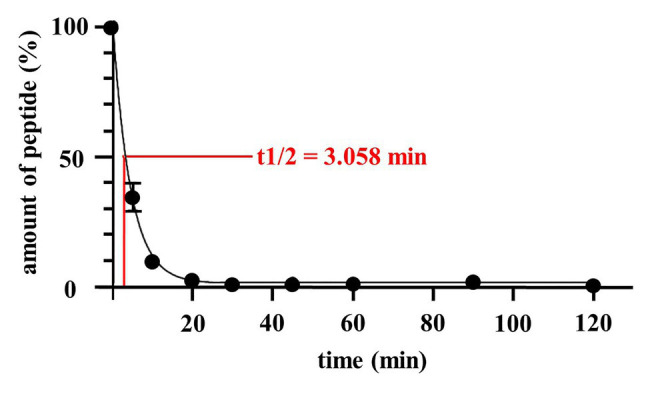
Half-life of Angie1 in human serum. Human serum was spiked with Angie1 (10 μM) and aliquots of the samples were harvested at indicated time points. The amount of peptide was determined by mass spectrometry. The graph shows the amount of peptide (%) of the initial inoculum ± SD of three individual measurements.

This prompted us to take initial steps toward developing Angie1 as a therapeutic agent to be evaluated in experimental models of tuberculosis infection. Along these lines, we measured the half-life in human serum, designed Angie1 containing liposomes as delivery vehicles and evaluated toxicity in an *in vivo* model. The half-life of Angie1 in human serum was 3.058 min as determined by mass spectrometry using an Orbitrap Elite System (Figure 8).

**Figure 9:**
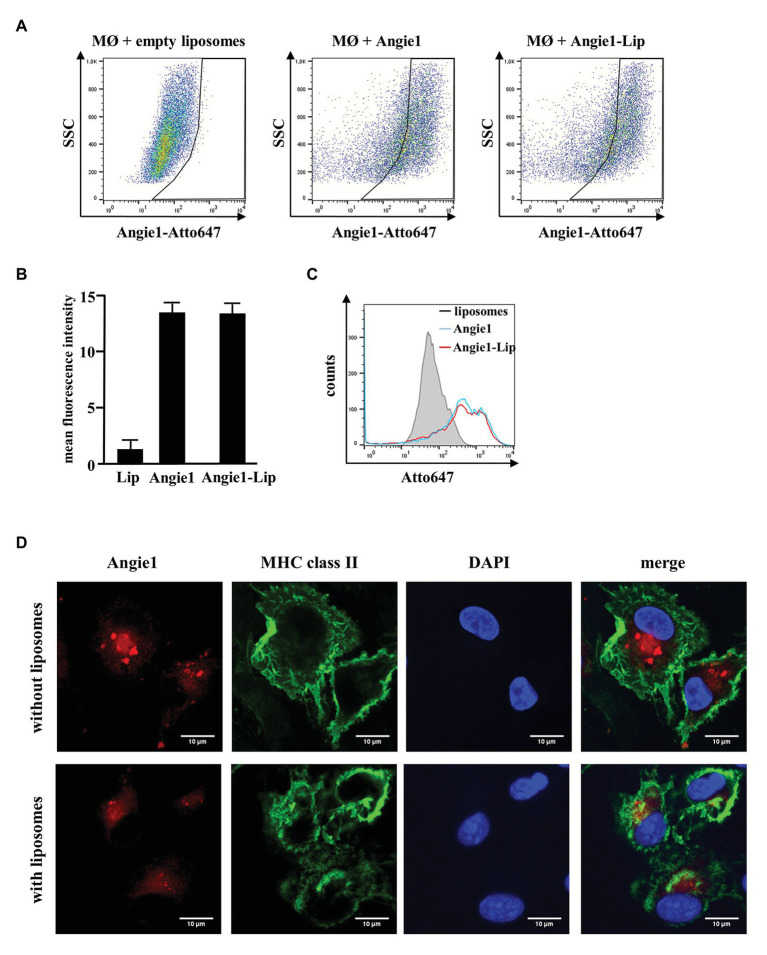
Uptake of Angie1 and Angie1-lip in macrophages. **(A)** Macrophages were incubated overnight with Atto647N-labeled Angie1 or Angie1-lip (both 54 μM, PSL Heidelberg). After 18 h, cells were harvested and analyzed by flow cytometry. Dot plots show one representative donor of three for each group. **(B)** The graph shows the mean fluorescence intensity (FI) ± SD of Atto647-positive cells for empty liposomes, Angie1 and Angie1-Lip of three independent donors. Statistical analysis was performed using a non-parametric Wilcoxon-Rank Test for paired samples. **(C)** The histogram shows the counts of Atto647-positive populations for empty liposomes, Angie1 and Angie1-Lip for one representative donor of three donors. **(D)** Macrophages were incubated with Angie1-Atto647N (upper panels) or Angie1-Atto647N-Lip (lower panels). After 18 h, cells were stained for MHC class II. Cell nuclei were stained with DAPI. Images were acquired using an inverted laser scanning confocal microscope (Zeiss LSM 710). Depicted images show representative area of one out of three donors with similar result.

One strategy to increase the stability of peptides *in vivo* is to deliver the compounds by liposomes, which gradually release the bioactive molecules. To evaluate whether this is an option for the delivery of Angie1, we labeled the peptide with the fluorescent dye Atto647N and incubated it with macrophages. Uptake of labeled and unlabeled Angie1 was determined by flow cytometry and confocal laser microscopy. Angie1 was readily incorporated into liposomes and yielded particles with a size of 190 nm (Supplementary Figure S2, left panel). Uptake of Angie1 by macrophages was equally efficient when delivered alone or incorporated into liposomes (Figures 9A–C). The intracellular localization of Angie1 in MHC class II positive macrophages was confirmed by confocal laser microscopy (Figure 9D). This demonstrates that liposomes provide an appropriate delivery platform for Angie1 and may be useful for increasing the stability of the peptide *in vivo*. Incorporation of Angiogenin into liposomes (Angiogenin-Lip) yielded particles with a size of 180 nm (Supplementary Figure S2, left panel). However, the delivery of Angiogenin-Lip into macrophages was not feasible due to the toxicity of the compound as evidenced by loss of adherence on plastic and nuclear condensation (Supplementary Figure S4).

**Figure 10:**
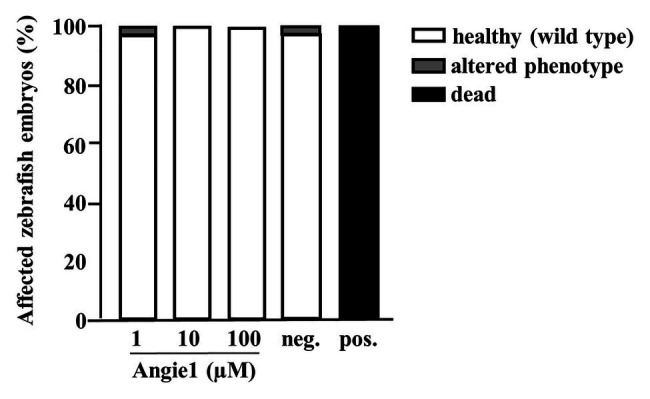
Angie1 is not toxic to zebrafish embryos. Zebrafish embryos were scored for mortality or altered phenotypes at 48 h post fertilization (hpf) after exposure for 24 h to Angie1 at the indicated concentrations or the negative control (PBS) or positive control [NRC-03 antimicrobial peptide, (AMP)]. Altered phenotypes include necrosis and non-lethal lysis (cytotoxicity), heart edema, reduced or absent circulation (cardiotoxicity), delayed development or malformations (developmental toxicity), and reduced or absent touch escape response (neurotoxicity). Note that Angie1 caused no significant toxicity. *n* = 60 embryos each group.

Zebrafish embryos provide a useful *in vivo* model for evaluating toxicity. We developed an experimental model, in which zebrafish embryos are exposed to a compound for 24 h, starting at 24 hpf, when most organ systems have already developed and are functional. Transparency of the embryos than allows for evaluation not only of mortality, but also for sublethal toxicity causing necrosis or lysis (acute toxicity/cytotoxicity), heart edema or reduced / absent circulation (cardiotoxicity), developmental delay or malformations (developmental toxicity), or reduced/absent touch escape response (neurotoxicity) under a light microscope. A standardized scoring system (Supplementary Figure S3A) together with the possibility of investigating embryos on a large scale, yields statistically solid and reproducible results. Angie1 in concentrations that demonstrated antimicrobial activity (1, 10, and 100 μM) showed no toxicity (Figure 10; Supplementary Figures S3B–D).

Taken together, our unbiased screen of a hemofiltrate peptide library identified a novel function of Angiogenin as an antimicrobial peptide with activity against *Mtb*. We designed a smaller derivative (Angie1), which maintains activity against *Mtb* can be efficiently delivered into human macrophages by liposomes and is not toxic for zebrafish embryos *in vivo*. Therefore, Angie1 is a novel AMP with favorable characteristics that is now ready to be tested for therapeutic efficacy in animal models of tuberculosis.

## Discussion

The application of antimicrobial peptides provides a novel concept for the treatment of infectious diseases, which fail to respond to conventional antibiotic treatment. By unbiased screening of a peptide library for activity against virulent *M. tuberculosis*, we identified Angiogenin as an antimicrobial compound in hemofiltrate. Activity against extracellular and intracellular *Mtb* was confirmed with synthesized Angiogenin. Using Angiogenin as a template, we generated a smaller peptide “Angie1” which could be delivered into *Mtb*-infected human macrophages by liposomes and limited the growth of the pathogen. These results highlight that (i) functional screening of human peptide libraries is a powerful approach to identify bioactive compounds, (ii) Angiogenin has antimicrobial activity, and (iii) *in silico* predictions can guide the optimization of lead components toward small, bioactive compounds with favorable toxicity and stability.

Angiogenin, also known as ribonuclease (RNase) 5, was first described as a potent inducer of vasculogenesis (Fett et al., 1985; Strydom et al., 1985). The peptide consists of 123 amino acids resulting in a molecular weight of 14.4 kDa. Mice express four Angiogenin genes, whereas there is only one gene (ANG) in humans (Cho et al., 2005). Angiogenin is secreted by multiple cell types, including macrophages, epithelial cells, and mast cells (Rybak et al., 1987). Release is increased in neurodegenerative diseases, malignancies, and inflammation (Sheng and Xu, 2016). Only one report associated human Angiogenin with antimicrobial activity against microbes, specifically *Enterococcus faecalis*, *Listeria monocytogenes*, *Streptococcus pneumoniae*, and *Candida albicans* (Hooper et al., 2003). The specificity of this finding was challenged because bovine serum albumin in the absence of Angiogenin had very similar effects (Avdeeva et al., 2006). Both studies were performed with recombinant Angiogenin expressed in *E. coli*. Our results strongly favor a specific activity of Angiogenin against *Mtb* as it was unequivocally identified by an unbiased approach from a hemofiltrate library and the activity was confirmed by using highly purified Angiogenin generated by solid-phase synthesis. Our experimental approach was designed to identify AMPs with activity against the major human pathogen *Mtb*, because tuberculosis is notoriously difficult to treat and only few antituberculotic drugs are available. Whether synthetic Angiogenin is also active against other microbial pathogens such as extracellular bacteria or fungi remains to be determined.

The route of delivery for AMPs to the site of infection remains a key challenge. Oral application is hampered by the catalytic cleavage in the acidic environment of the stomach. Intravenous infusion will expose the peptide to an unfavorable environment in the serum with high concentrations of salt and digestive enzymes. In addition, the synthesis of sufficient quantities of peptide required for *in vivo* application is costly. These drawbacks are directly related to the size of the peptide. Restriction of the peptide length to regions directly required for the bioactivity would be highly advantageous. Therefore, we applied *in silico* bioinformatic tools to screen databases of antimicrobial sequences to identify the antimicrobial region. The candidate sequence was further modified to increase the positive charges required for the interactions with the mycobacterial cell wall. The resulting peptide Angie1 (123aa®16aa; molecular weight 14.1 kDa®1.9 kDa) can easily be produced in large quantities, is accessible to further modifications and importantly maintains activity against virulent *Mtb* in the same order of magnitude as the parental Angiogenin. We do not provide experimental evidence that Angie1 is the most active region of Angiogenin. We took an *in silico* approach to identify the potential cytolytic region. The alternative and possibly more definitive approach would have been to synthesize overlapping peptides. As cellular biologists, we preferred to take the hypothesis-driven approach and will now improve uptake and activity by engineering delivery vehicles such as liposomes (Figure 9) or mesoporous particles. Even though the activity of Angie1 against extracellular *Mtb* is lower as compared to Angiogenin the abovementioned benefits regarding the potential stability, specificity and availability make Angie1 the superior candidate for further evaluation in *in vivo* models of infectious diseases.

The application of AMPs in tuberculosis or other systemic infectious diseases would be in combination with-rather than replacing-conventional antibiotic treatment. In principle AMPs could be combined with (i) conventional tuberculosis drugs in accordance with drug susceptibility testing, (ii) adjuvants which promote effector mechanisms of macrophages, or (iii) molecules that optimize the targeting of AMPs to the site of infection. One approach to achieve this goal is the design of multipurpose nanoparticles, which serve as a delivery vehicle for multiple bioactive molecules (Singh, 2019). Toward this goal, we initially generated liposomes containing a DDB backbone, the adjuvant trehalose dibehenat (TDB), and Angie1. TDB is synthetic derivative of the mycobacterial cord factor and binds to Mincle (Ishikawa et al., 2009), thereby supporting the activation of macrophages (Ostrop et al., 2015; Huber et al., 2020). These liposomes (CAF01) are well established for the delivery of vaccine antigens and have increased the protective efficacy of subunit vaccines against tuberculosis (Agger et al., 2008; van Dissel et al., 2014). The biochemically related mycobacterial cell wall component muramyl dipeptide packed into liposomes is already a licensed adjuvant treatment for osteosarcoma (Kleinerman et al., 1992). Such liposomes provide a versatile platform and can be loaded with additional adjuvants, for example, to trigger Toll like receptor-mediated antimicrobial pathways (Kennerknecht et al., 2020). Angie1-containing liposomes were efficiently taken up by macrophages (Figure 9) and will provide the starting point for designing optimized nanodrug delivery systems, for example, by including SIGLEC-7 to support specific targeting of *Mtb*-infected macrophages (Kawasaki et al., 2014). In addition, liposomes support storage and gradual release of the incorporated compounds in tissue (Simão et al., 2015). Another beneficial effect of a liposomal delivery system is that uptake and clearance of the active compound by macrophages can be reduced or avoided (Li et al., 2019). By inference, we hypothesize that delivery of Angie1 by liposomes will extend the half-life of Angie1 in human serum and tissue beyond the 3 min measured for free Angie1 (Figure 8).

Another promising delivery platform are mesoporous nanoparticles, which have been used to deliver antimicrobial peptides, such as NZX and LL-37 (Braun et al., 2016; Tenland et al., 2019) to enhance protection against tuberculosis in mice (Tenland et al., 2019). Nanoparticles also promote stability, bioavailability, and efficacy of conventional antituberculotic drugs (Clemens et al., 2012; Singh, 2019). These studies will guide the development of multipurpose nanoparticles combining Angie1, antibiotics, and adjuvants to achieve optimized antimicrobial activity at the site of infection.

Only few AMPs with activity against virulent *Mtb* have been described, which may be related to the complexity of the lipid-rich mycobacterial cell wall, the slow metabolism/generation time of *Mtb* or simply due to the requirements for a biological safety level 3 laboratory. Granulysin (Stenger et al., 1998), human neutrophil peptides, such as human ß-defensins (Linde et al., 2001), Protegrin 1 (Fattorini et al., 2004), Lipocalin 1 (Martineau et al., 2007), LL-37 (Deshpande et al., 2020), Lassomycin (Gavrish et al., 2014), Teixobactin (Ling et al., 2015), or Hepcidin (Sow et al., 2007) act at fairly high concentrations ranging between 10 and 50 μM, which is within the same range as we observed for Angiogenin and Angie1 (Figures 2, 5). Generally, cationic AMPs interact with the negatively charged mycomembrane and plasma membrane of *Mtb* (Gutsmann, 2016). Angiogenin, which has a net charge of +10 is also highly cationic and we hypothesize that it will also interact with the mycomembrane. Accordingly, we modified the predicted cytotoxic region of Angiogenin (aa 64–80) to increase the positive net charge (+3) which resulted in higher antibacterial activity, even though, we do not provide proof that there is an interaction with the mycobacterial cell wall. Whether the disruption of the mycobacterial cell wall by AMPs is already sufficient to kill the bacilli or only represents the first step of an antimicrobial pathway remains to be determined. Since Angiogenin belongs to the ribonuclease A (RNase A) superfamily, the catalytic activity could contribute to the degradation of bacterial RNA and cause lethal damage to the pathogen (Ganz, 2003). Other possible effector mechanisms include the inhibition of cell wall synthesis, the interference with the mycobacterial iron metabolism or the targeting of ATP-dependent proteases (Gutsmann, 2016). In the case of intracellular *Mtb*, the direct effect on the bacteria could be supported by AMP-mediated modulation of macrophage function. LL-37, Defensins (Hancock et al., 2016), and Granulysin (Deng et al., 2005; Tewary et al., 2010) affect chemotaxis and cytokine responses by macrophages. While Angiogenin or Angie1 do not induce chemotaxis or cytokine release by primary human monocytes or macrophages (not shown) it remains to be determined whether there is an immune-modulatory effect on *Mtb*-infected macrophages.

In conclusion, we detected Angiogenin as an endogenous antimycobacterial peptide in human serum. Using Angiogenin as a template, we engineered the small, biologically active derivative Angie1 with activity against virulent *Mtb* and fast-growing bacteria. Angie1 can be efficiently delivered into human macrophages *via* liposomes, providing the intriguing perspective of testing the therapeutic efficacy in small animal models of infection. The combined application of peptidomics, *in silico* prediction of biological function, nanomedicine, and microbiology is a powerful multidisciplinary approach for the discovery of novel antimicrobial compounds.

## Data Availability Statement

The raw data supporting the conclusions of this article will be made available by the authors, without undue reservation, to any qualified researcher. Proteomic datasets are available at the following link: http://massive.ucsd.edu/ProteoSAFe/status.jsp?task=b7605ed4d7b74921b49260d202cd0103.

## Author Contributions

RN and FG performed experiments with *Mtb* and edited the manuscript. RN performed flow cytometry and confocal microscopy. FG helped with flow cytometry and data analysis. JK, DM, and MG helped with *Mtb* assays. FL produced and characterized liposomes. AH performed assays with fast-growing bacteria under supervision of BS. MR and GW investigated cytotoxicity against zebrafish embryos. W-GF provided the hemofiltrate library and AR with LS purified Angiogenin. SW and AR performed mass spectrometry analysis and determined half-life in serum. SS designed all experiments, supervised the study, validated the data, and wrote the manuscript. All authors contributed to the article and approved the submitted version.

### Conflict of Interest

W-GF was employed by Pharis Biotec GmbH.

The remaining authors declare that the research was conducted in the absence of any commercial or financial relationships that could be construed as a potential conflict of interest.

## Abbreviations

AMP: Antimicrobial peptide
BSA: Bovine serum albumin
CFU: Colony forming units
GM-CSF: Granulocyte-macrophage colony-stimulating factor
MOI: Multiplicity of infection
*Mtb*: *Mycobacterium tuberculosis*
PBMC: Peripheral blood mononuclear cell
PFA: Paraformaldehyde
TFA: Trifluoroacetic acid
