# Supplementary material for: Unbiased Identification of Angiogenin as an Endogenous Antimicrobial Protein With Activity Against Virulent Mycobacterium tuberculosis
Source: Front Microbiol. 2021 Jan 18;11:618278. doi: 10.3389/fmicb.2020.618278 (PMC7848861; doi:10.3389/fmicb.2020.618278)
Supplement: Supplementary file 1 [file Data_Sheet_1.docx]

Supplementary Material


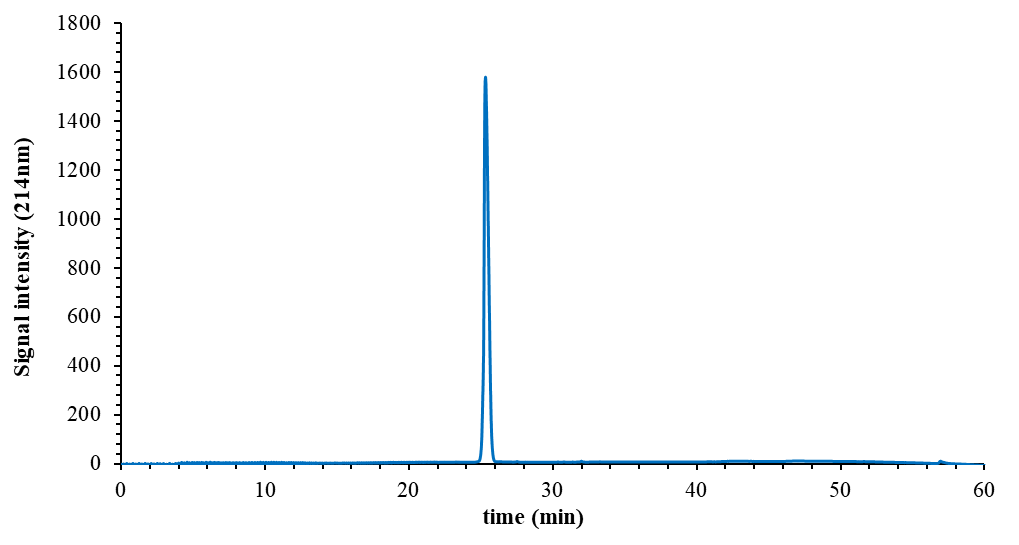


**Supplementary Figure 1.** **Reversed-phase C18 HPLC analysis of synthetic Angie1**

Angie1 was injected into a Jupiter reversed-phase C18 HPLC column (Phenomenex, CA, USA) of dimensions 4.6 x 25 cm, particle size 5 µm, pore size 300 Å and measured at a flow rate of 1 ml/min. The gradient program was (min/%B): 0/5 45/50 60/80 and the elution was UV-monitored by HPLC Agilent 1100 (Agilent Technologies, Santa Clara, USA) at 214 nm.


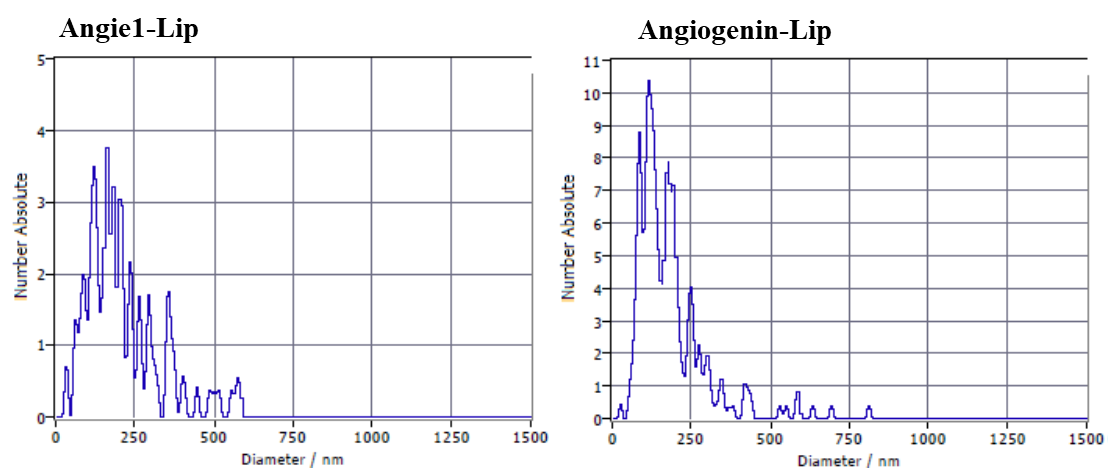


**Supplementary Figure 2.** **Size and concentration of Angie1-Lip and Angiogenin-Lip**

Angie1-Lip and Angiogenin-Lip were analyzed by nanoparticle tracking analysis using ZetaView Analyze (Version 08.05.05 SP2). The graph shows one representative measurement of six.


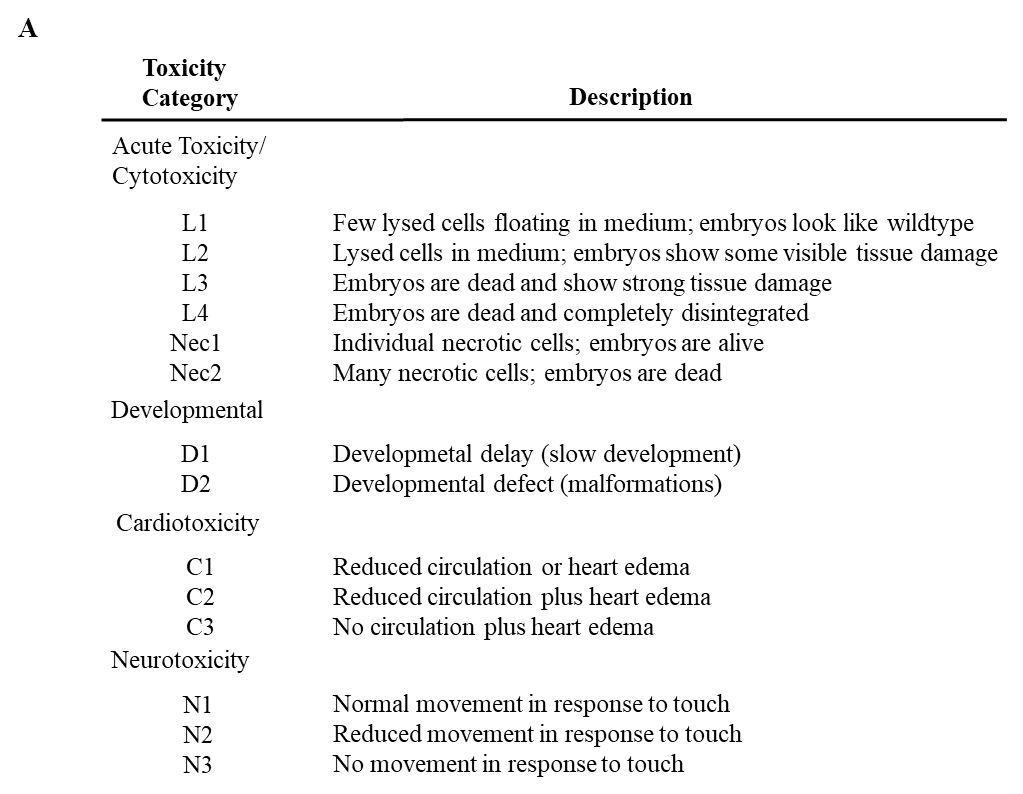

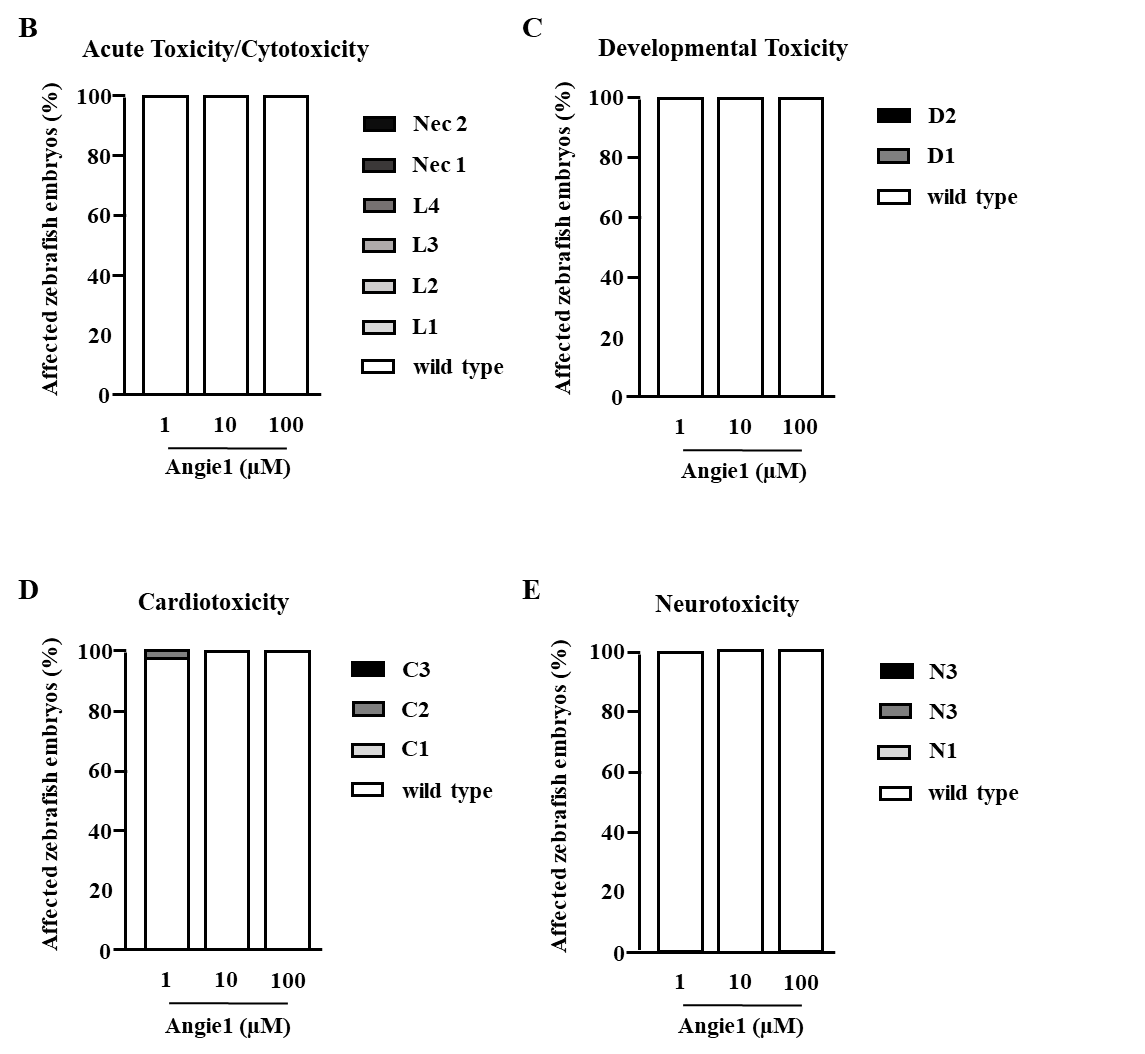


**Supplementary Figure 3.** **Toxicity scoring system in zebrafish embryos.**

Embryos were classified into the listed categories based on the phenotypes described in the right column. n = 60 embryos each group.


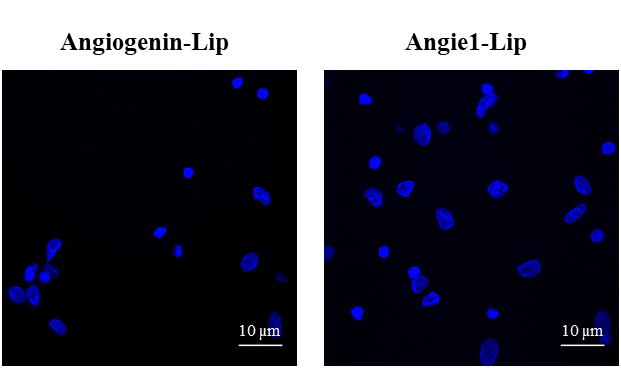


**Supplementary Figure 4. Effect of Angiogenin-Lip or Angie1-Lip on macrophages.**

Human macrophages were incubated with Angiogenin-Lip or Angie-Lip for 18 hrs. Cell nuclei were stained with DAPI. The pictograms show representative images from one representative donor (n=3).
